# Corticospinal excitability of tibialis anterior and soleus differs during passive ankle movement

**DOI:** 10.1007/s00221-019-05590-3

**Published:** 2019-06-26

**Authors:** Jakob Škarabot, Paul Ansdell, Callum G. Brownstein, Kirsty M. Hicks, Glyn Howatson, Stuart Goodall, Rade Durbaba

**Affiliations:** 1 Faculty of Health and Life Sciences, Northumbria University, Newcastle upon Tyne, England NE1 8ST UK; 2 Univ Lyon, UJM-Saint-Etienne, Laboratoire Interuniversitaire de Biologie de la Motricité, EA 7424, 42023 Saint-Étienne, France; 3 Water Research Group, School of Environmental Sciences and Development, Northwest University, Potchefstroom, South Africa

**Keywords:** Ia afferent, Fascicle length, *H*-reflex, Transcranial magnetic stimulation

## Abstract

The purpose of this study was to assess corticospinal excitability of soleus (SOL) and tibialis anterior (TA) at a segmental level during passive ankle movement. Four experimental components were performed to assess the effects of passive ankle movement and muscle length on corticospinal excitability (MEP/*M*_max_) at different muscle lengths, subcortical excitability at the level of lumbar spinal segments (LEP/*M*_max_), intracortical inhibition (SICI) and facilitation (ICF), and *H*-reflex in SOL and TA. In addition, the degree of fascicle length changes between SOL and TA was assessed in a subpopulation during passive ankle movement. Fascicles shortened and lengthened with joint movement during passive shortening and lengthening of SOL and TA to a similar degree (*p* < 0.001). Resting motor threshold was greater in SOL compared to TA (*p* ≤ 0.014). MEP/*M*_max_ was facilitated in TA during passive shortening relative to the static position (*p* ≤ 0.023) and passive lengthening (*p* ≤ 0.001), but remained similar during passive ankle movement in SOL (*p* ≥ 0.497), regardless of muscle length at the point of stimulus (*p* = 0.922). LEP/*M*_max_ (SOL: *p* = 0.075, TA: *p* = 0.071), SICI (SOL: *p* = 0.427, TA: *p* = 0.540), and ICF (SOL: *p* = 0.177, TA: *p* = 0.777) remained similar during passive ankle movement. *H*-reflex was not different across conditions in TA (*p* = 0.258), but was reduced during passive lengthening compared to shortening in SOL (*p* = 0.048). These results suggest a differential modulation of corticospinal excitability between plantar and dorsiflexors during passive movement. The corticospinal behaviour observed might be mediated by an increase in corticospinal drive as a result of reduced afferent input during muscle shortening and appears to be flexor-biased.

## Introduction

Corticospinal excitability is constantly modulated during passive and active movements. Isotonic movements modify corticospinal excitability, such that excitability tends to be lower during lengthening relative to shortening and isometric contractions (Abbruzzese et al. 1994; Gruber et al. 2009; Duclay et al. 2014), which seems to depend on the amount of Ia afferent feedback (Doguet et al. 2017). However, elucidating the direct effect of muscle length-related feedback on the corticospinal tract output during dynamic contractions is challenging due to the influence of postsynaptic control mechanisms (Valadão et al. 2018; Barrué-Belou et al. 2018), and potential differences in neural drive that can influence neurophysiological responses (Abbruzzese et al. 1994; Morita et al. 2000).

Potential insight into the effect of muscle length-related feedback on the corticospinal response might be gained by assessing responses during passive movement. With passive muscle lengthening, the firing of muscle spindle afferents increases proportionally to the magnitude of the stretch, but remains low during shortening of a muscle (Matthews 2011; Day et al. 2017). This behaviour at the somatosensory receptor level might, in turn, modulate the corticospinal responses. Indeed, corticospinal excitability has been shown to be reduced during passive lengthening of the wrist flexors and extensors, and has been related to the degree of muscle spindle afferent feedback (Lewis et al. 2001; Lewis and Byblow 2002; Coxon et al. 2005). Notwithstanding these findings, the level of neural axis at which afferent-mediated changes in corticospinal output occur has not been elucidated. From a cortical perspective, intracortical inhibition is modulated during passive shortening and lengthening of the upper limbs (Lewis et al. 2001). However, despite the presence of a facilitatory corticospinal response during passive shortening of upper limb muscles (Chye et al. 2010), the contribution of intracortical facilitatory circuits to augmented corticospinal excitability has not been considered. In addition, passive lengthening of soleus (SOL) has been shown to be accompanied by greater presynaptic inhibition (Pinniger et al. 2001), whilst less is known about the effect of passive movement on subcortical output of the corticospinal tracts, which are likely devoid of classical presynaptic influence (Nielsen and Petersen 1994). In addition, far less is known about corticospinal excitability during passive movement of the lower limbs, which might differ due to disparities between facilitatory and inhibitory intracortical outputs and corticospinal projections to upper and lower limb muscles (Brouwer and Ashby 1990; Chen et al. 1998).

The SOL and tibialis anterior (TA) muscles are integral for movement about the ankle joint. For example, SOL plays a crucial role in balance (Capaday et al. 1999), whereas TA is involved in the control of foot drop during heel strike and foot lift during the swing phase (Byrne et al. 2007), as well as toe clearance through the gait cycle (Nielsen et al. 2003). The SOL and TA muscles also exhibit distinct roles in quiet standing and postural sway, with the former acting as agonist and the latter providing the proprioceptive feedback via reciprocal inhibition (Di Giulio et al. 2009). Due to these differences in function, TA and SOL might require distinct corticospinal control. From a neural perspective, TA and SOL have been shown to exhibit differences in the quantity of muscle spindles that affects the relative input from Ia afferents (Banks 2006; De Luca and Kline 2012), the type and the size of motor units (Burke 1967; Dum and Kennedy 1980), reciprocal spindle afferent input (Yavuz et al. 2018), distribution of direct corticomotoneuronal projections (Brouwer and Ashby 1992; Brouwer and Qiao 1995), intracortical inhibition (Lauber et al. 2018), and preferences in the input from pyramidal tract into the spinal network (Brooks and Stoney 1971).

The aim of this study was to investigate corticospinal function of TA and SOL during passive ankle movement. Four experimental components were performed designed to assess (1) corticospinal modulation at different muscle lengths; (2) the contribution of cortical neurons and spinal motoneurons to the corticospinal response: (3) intracortical facilitation and inhibition; and (4) the contribution of Ia afferent input to spinal motoneurons in quiescent SOL and TA during passive ankle movement. It was hypothesised that corticospinal excitability will be dependent on the change in muscle length and muscle studied, and will be attributable to processes at both cortical and spinal levels.

## Methods

### Participants

Twenty healthy, volunteers (25 ± 4 years, 175 ± 9 cm, 78.9 ± 16.8 kg; 9 females) participated in the study. Based on the previous studies (Lewis et al. 2001; Lewis and Byblow 2002), an a priori power analysis (Faul et al. 2007) showed that six participants were needed to observe modulation of MEP amplitude with passive movement. To reduce the potential influence of female sex hormones on TMS-evoked responses, all females were tested in the early follicular phase of the menstrual cycle where both oestrogen and progesterone concentrations are likely to be low (Elliott et al. 2003) or whilst taking oral contraceptives (Ansdell et al. 2019). All participants were free from neurological illness or musculoskeletal injury, were not taking any medications known to affect the nervous system, and reported no contraindications in TMS safety screening (Keel et al. 2001). The study conformed to the standards of Declaration of Helsinki, apart from pre-registration in a database. All procedures were approved by Northumbria University Ethics Committee (BMS57UNNJSRD2016). All participants provided written informed consent prior to the start of the study proceeding.

### Experimental design

The study involved four experimental components designed to investigate the effect of passive ankle motion on corticospinal excitability at different muscle lengths (Experiment 1), corticospinal and spinal motoneuron excitability (Experiment 2), intracortical facilitation and inhibition (Experiment 3), and the contribution of Ia afferent input to spinal motoneurons (Experiment 4) in resting SOL and TA. Twelve participants took part in Experiment 1 (26 ± 4 years, 176 ± 9 cm, 77.8 ± 16.8 kg; 6 females). In Experiment 2, two participants did not return for further testing due to scheduling conflicts, and an additional participant was recruited (*n* = 11; 26 ± 4 years, 178 ± 8 cm, 81.6 ± 16.2 kg; 5 females). Due to larger heterogeneity of responses, additional participants were recruited for Experiment 3 (*n* = 15; 25 ± 4 years, 178 ± 9 cm, 83.1 ± 17.1 kg; 5 females). In Experiment 4, obtaining *H*-reflexes in resting TA proved challenging as has been previously reported (Roy and Gorassini 2008; Burke 2016). After screening 24 individuals, only five participants exhibited clear and consistent *H*-reflexes in quiescent TA to allow for comparison with SOL and took part in Experiment 4 (24 ± 3 years, 176 ± 11 cm, 72.2 ± 14.3 kg; 1 female). Individuals that took part in all four experiments were tested within 6 weeks of the first visit to the laboratory.

### Procedures

#### Experimental setup

Participants sat on an isokinetic dynamometer (Cybex, Lumex Inc., USA) with hip and knee at 60° and 90° flexion, respectively. All testing was performed on the dominant limb as determined by the lateral preference inventory (Coren 1993). The foot was strapped securely to a metal foot plate attached to the lever arm of the motor with a velcro strap. The range of motion of the device was set to 20°, ranging from 10° plantar flexion to 10° dorsiflexion with anatomical zero being when the ankle was set at 90°. During passive ankle motion, the motor of the device moved the foot plate throughout the range of motion at 5° s^−1^. TMS or electrical stimulation was delivered at anatomical zero (considered intermediate muscle length) during static position and passive ankle movement. In addition, stimuli were delivered at ± 7.5° relative to anatomical zero in the part of the study examining corticospinal responses at different muscle lengths during passive ankle movement, with positive and negative degree values indicating plantar and dorsiflexion, respectively. Thus, at positive values relative to anatomical zero, the muscle was at longer and shorter length for TA and SOL, respectively, and vice versa for negative values. Based on the joint angles and movement velocity, the stimuli were delivered 2 s (Experiment 1–4), and 0.5 and 3.5 s after the onset of movement (Experiment 1). To minimise thixotropic effect on the responses, participants were resting in the starting position at least 10 s before the start of passive motion (Proske et al. 1993). At least 15 s of rest was employed before each motion.

#### Electromyography

Electromyographic (EMG) activity was recorded with a bipolar electrode arrangement (8 mm diameter, 20 mm inter-electrode distance; Kendall 1041PTS, Tyco Healthcare Group, USA) over the muscle belly of SOL and TA with the reference electrode placed over the medial malleolus according to SENIAM recommendations (Hermens et al. 2000). For SOL, the electrodes were positioned at two-thirds of the line between the medial condyle of the femur to the medial malleolus. For TA, the electrodes were placed at one-third of the length between the tip of the fibula and the tip of the medial malleolus. Prior to placement of electrodes, the recording site was shaved, abraded with preparation gel, and wiped clean with an alcohol swab to ensure appropriate impedance (< 2 kΩ). The EMG signal was amplified (1000×), band pass filtered (20–2000 Hz; Neurolog System, Digitimer Ltd, UK), digitised (5 kHz; CED 1401, CED, UK), acquired, and analysed off line (Spike2, v8, CED, UK).

#### Transcranial magnetic stimulation

Single- and paired-pulse TMS were delivered using two Magstim 200^2^ magnetic stimulators (Magstim Co., Ltd., Whitland, UK) via a concave double-cone coil. The coil was positioned over the leg area of the primary motor cortex contralateral to the target dominant leg and was oriented to induce posterior-to-anterior cortical current. Whilst corticospinal responses might differ between the dominant and non-dominant hemisphere in the upper limbs, evidence is lacking that a similar difference exists for lower limbs (Smith et al. 2017). Initially, the centre of the coil was placed 1 cm lateral and posterior to the vertex (Devanne et al. 1997), after which it was moved medio-laterally and posterior-anteriorly in small steps around the initial position until the spot consistently evoking the greatest MEP in the target muscle, i.e., SOL or TA, was identified (hotspot). Once identified, the back of the coil was marked directly on the scalp to ensure consistent placement throughout the trial. Resting motor threshold (rMT) was then established with the ankle positioned at anatomical zero and determined as the intensity that elicited an MEP amplitude ≥ 50 µV in 3 out of 5 trials (Rossini et al. 1994). The hotspot and rMT were determined separately for SOL and TA, and separately during each experimental session.

#### Lumbar-evoked potentials

Lumbar-evoked potentials (LEPs) were elicited with a constant-current stimulator (1 ms pulse duration; Digitimer DS7AH, Hertfordshire, UK) to assess spinal motoneuronal excitability during passive movement of the ankle. The cathode was centred over the first lumbar spinous process (5 × 9 cm; Nidd Valley Medical Ltd., Bordon, UK) with the long axis of the electrode aligned to the centre of the vertebral column. The surface area of the cathode covered two spinous processes above and below the centre point (*T*_11_–*L*_3_). A cathode of large area was chosen as it produced less discomfort and greater tolerance by participants (Ugawa et al. 1995; Kuhn et al. 2010). The anode (2.5 cm^2^) was placed 5 cm above the upper edge of the cathode (Ugawa et al. 1995), corresponding to the level of the eighth thoracic spinous process (*T*_8_). This stimulating site has recently been shown to activate corticospinal axons at the level of lumbar spinal segments (Škarabot et al. 2018).

#### Percutaneous nerve stimulation

Percutaneous nerve stimulation (1 ms pulse duration; Digitimer DS7AH, Hertfordshire, UK) was performed to elicit *H*-reflexes in Experiment 4 in SOL and TA (see ‘Experiment 4’ for a more detailed procedure). To account for changes at the skin–electrode surface, maximal compound action potentials (*M*_max_) were elicited in SOL and TA and subsequently used for normalisation of the responses across Experiments 1–4. To evoke responses in SOL, the cathode (2.5 cm^2^; Nidd Valley Medical Ltd., Bordon, UK) was placed over the tibial nerve in the popliteal fossa with the anode (5 × 9 cm) positioned over the patella. To elicit responses in TA, a 40 mm cathode/anode arrangement (Digitimer, Hertfordshire, UK) was placed over the common peroneal nerve below the head of the fibula. *M*_max_ was elicited separately for SOL and TA by gradually increasing the intensity of percutaneous stimulation until the EMG response plateaued, upon which the intensity was further increased by 30%. In Experiments 1–4, four stimuli eliciting *M*_max_ in both muscles were delivered at anatomical zero. In addition, in Experiment 1, four *M*_max_ were elicited at ± 7.5° relative to anatomical zero. Since *M*_max_ is sensitive to changes in static positions (Gerilovsky et al. 1989), but not shortening and lengthening when stimuli are delivered at the same joint angle (Pinniger et al. 2001), *M*_max_ was elicited only during static positions.

### Experimental procedures

**Fig. 1:**
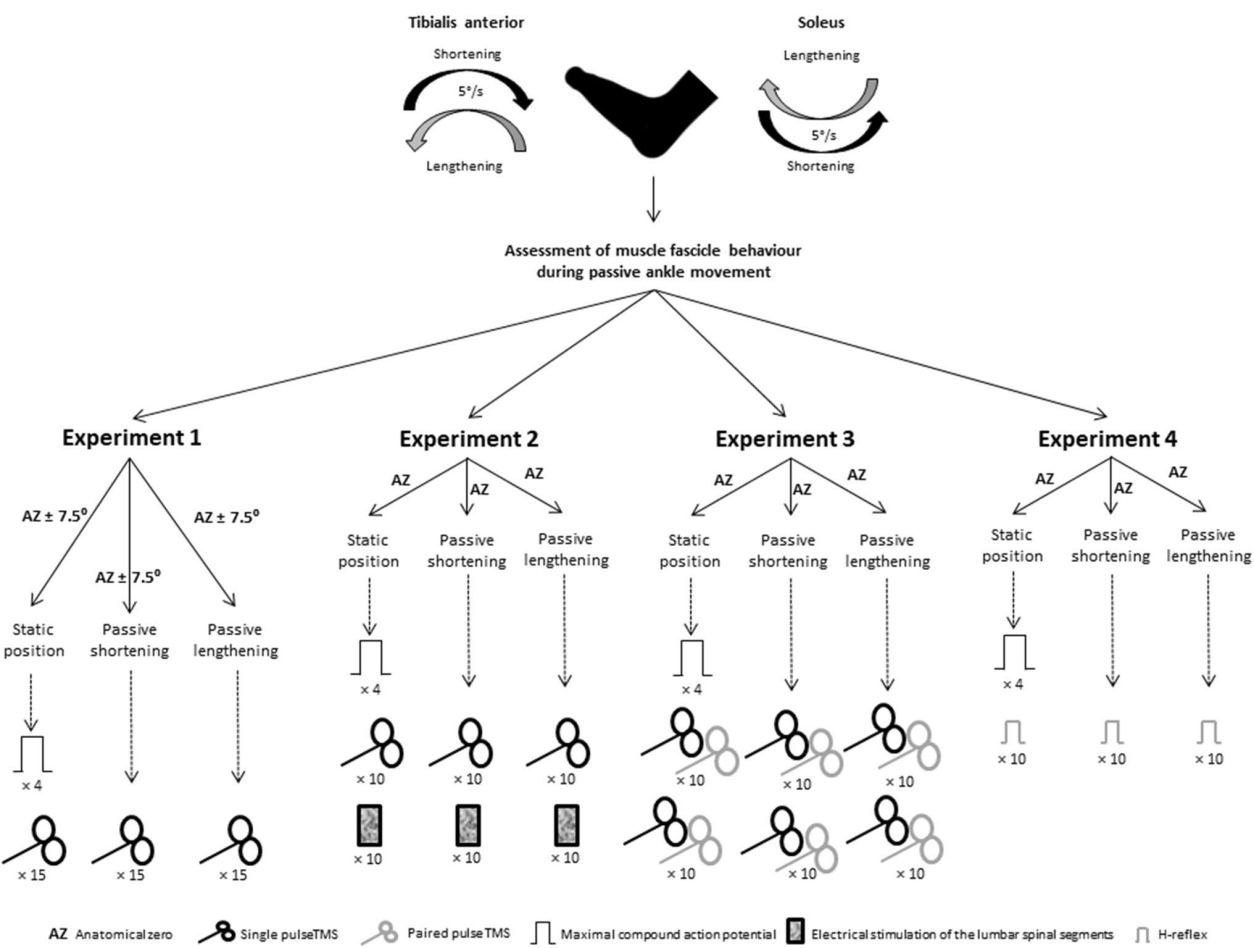
An overview of experimental procedures

The experimental procedures are summarised in Fig. 1.

#### Assessment of fascicle length changes during passive ankle movement

Changes in joint angle during passive movement of muscle are usually assumed to reflect changes in the total muscle–tendon unit length. However, the proprioceptive feedback originating from muscle spindles is more closely related to changes in fascicle length than joint angle (Matthews and Stein 1969; Morgan et al. 2000; Day et al. 2017). As modulation of corticospinal excitability has been linked to afferent feedback pertaining to changes in muscle length (Lewis et al. 2001; Lewis and Byblow 2002; Coxon et al. 2005), it is important to establish whether changes in joint angle correspond to changes in fascicle length. Furthermore, it is important to assess the similarity of those changes between TA and SOL to ensure that the corticospinal responses are not confounded by differing magnitude of afferent feedback between the two muscles.

**Fig. 2:**
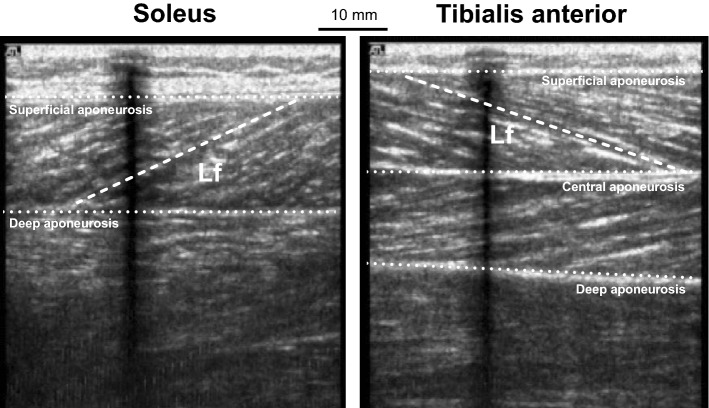
An example of ultrasound sagittal plane scans. Images were taken at anatomical zero and show the fascicle length (Lf) measured from the visible insertion of the fibre between the deep and superficial aponeurosis in soleus (left panel), and from central to the superficial aponeurosis tibialis anterior (right panel). The shadow in the images represents the echo-absorptive marker used to ensure no movement between the skin and the probe occurred throughout ankle movement

In a subpopulation of seven individuals (27 ± 3 years, 179 ± 8 cm, 84.1 ± 19.6 kg; 3 females), fascicle behaviour of the SOL and TA during 20° of passive ankle movement at 5° s^−1^ was tracked using ultrasound. Ultrasound (AU5 Harmonic, Esatoe Biomedica, Genoa, Italy) images were captured in real time (25 Hz sampling; AVer Media Capture Studio, AVer Media Technologies, New Taipei City, Taiwan). After identification and marking of the proximal and distal insertion of the muscle, a B-mode linear array probe (7.5 MHz, 55 mm width) was held with constant light pressure, perpendicular to the dermal surface along the midsagittal plane of the muscle. For SOL, the probe was positioned at 50% of the distance between the popliteal crease and the lateral malleolus (Valadão et al. 2018). In three participants, this position had to be adjusted to 30% of the same reference line to allow for clear imaging of the fascicles (Valadão et al. 2018). For TA, the probe was positioned between the fibular head and medial malleolus (Bland et al. 2011) at the site corresponding to the thickest portion of the muscle as identified by the ultrasound (Reeves and Narici 2003). A hypo-allergenic ultrasound gel (Parker, Park Laboratories Inc., Fairfield) was used to enhance coupling between the skin and the probe. An echo-absorptive marker was placed between the skin and the probe to ensure the probe did not move during the recording. An externally generated square wave pulse was used to synchronise the ultrasound images with the dynamometer position acquisition system. Frame-capture software (Adobe Premier Elements, version 15) was used to acquire ultrasound images, corresponding to every 0.5° of ankle angle, for offline analysis. Using digitising software (ImageJ 1.45, National Institutes of Health, USA), SOL and TA fascicle length was measured at full ROM (± 10° relative to anatomical zero) and the positions corresponding to where stimulations were delivered (anatomical zero and ± 7.5° relative to anatomical zero). Fascicle length was measured from the visible insertion of the fibre between the deep and superficial aponeurosis for SOL (Valadão et al. 2018), and from central to the superficial aponeurosis for TA (Fig. 2; Reeves and Narici 2003). The fascicle was measured if it remained visible across the entire ultrasound image. Where the fascicle extended beyond the ultrasound image, linear continuation of the fascicle and aponeurosis was assumed (ICC = 0.853, Ando et al. 2014; 2.4% error rate, Reeves and Narici 2003). To reduce error associated with estimation of fascicle length, an average of three fascicles across the image was taken (Guilhem et al. 2011).

#### Experiment 1: corticospinal responses at different muscle lengths during passive ankle movement

Responses in 12 individuals were assessed across nine conditions: static position and passive shortening and lengthening with single-pulse TMS delivered at anatomical zero (intermediate muscle length) and at ± 7.5° relative to anatomical zero (shorter and longer muscle length depending on the muscle as explained above). The order of conditions was randomised. Intensity of TMS was standardised to 1.2 × rMT in the static position, as this intensity corresponds with the ascending limb of the stimulus–response curve (Han et al. 2001), making the responses susceptible to changes with passive ankle movement. A total of 15 MEPs were elicited in each condition.

#### Experiment 2: corticospinal and spinal motoneuronal responses during passive ankle movement

In eleven individuals, ten LEPs and ten MEPs were evoked during static position and passive ankle movement in SOL and TA (randomised order). The intensity of TMS was standardised to 1.2 × rMT. Pilot testing indicated that MEPs elicited at 1.2 × rMT in the resting position evoke a response of ~ 5–10% *M*_max_. Thus, the stimulus intensity of LEPs was standardised to elicit a response of ~ 5–10% *M*_max_ in the resting position (current intensity: 151 ± 54 and 163 ± 54 mA for SOL and TA, respectively). All stimuli were delivered at anatomical zero.

#### Experiment 3: intracortical inhibition and facilitation during passive ankle movement

In 15 participants, paired-pulse paradigms (SICI and ICF) were employed during static position and passive movement of the ankle to elicit responses in SOL and TA (randomised order). The TMS configuration used consisted of conditioning stimuli of 0.7 and 0.6 × rMT and ISIs of 2 and 10 ms for SICI and ICF, respectively (Brownstein et al. 2018). The test stimulus was always delivered at 1.2 × rMT. Ten unconditioned and ten conditioned pulses were delivered in an alternating fashion for each paired-pulse paradigm at anatomical zero.

#### Experiment 4: *H*-reflex during passive ankle movement

In five participants, *H*/*M* recruitment curves were first constructed in the anatomical zero position in both SOL and TA by gradually increasing the intensity of stimulation by 0.3 mA every three pulses from *H*-reflex threshold to *M*_max_. Recruitment curves were obtained only in the static position since only the amplitude of the *H*-reflex, but not the slope of the *H*/*M* curve differs between passive shortening and lengthening (Pinniger et al. 2001). The *H*-reflex amplitude was evoked with a small *M*-wave of consistent size across conditions (SOL: 12 ± 6% *M*_max_, TA: 8 ± 2% *M*_max_; *p* = 0.21), ensuring that the same proportion of motor units were activated across conditions (Duclay and Martin 2005), and that the *H*-reflex was produced on the ascending limb of the *H*/*M* recruitment curve and was, thus, susceptible to a change with passive ankle movement (Pierrot-Deseilligny and Burke 2005). Ten *H*-reflexes were elicited in SOL and TA during static position and passive ankle movement in a randomised order. All stimuli were delivered at anatomical zero. Recordings were made separately for TA and SOL.

### Data analyses

EMG activity was visually inspected during the experiments to ensure that participants maintained a relaxed muscle. If voluntary EMG activity was observed, the trial was discarded and additional trials were performed. Furthermore, root-mean-square EMG activity (RMS_EMG_) was measured 100 ms prior to each stimulus to ensure that participants were relaxed. If RMS_EMG_ was > 2 standard deviations (SD) compared to mean baseline values, the evoked response following it was discarded. For that reason, SICI and ICF data from one participant were omitted from statistical analysis. RMS_EMG_ data across all conditions and experiments are displayed in Table 1. Peak-to-peak amplitudes of the evoked responses were calculated. MEPs, LEPs and *H*-reflex peak-to-peak amplitudes were expressed as a percentage of peak-to-peak amplitudes of *M*_max_ (MEP/*M*_max_, LEP/*M*_max_, and *H*/*M*_max_, respectively). To quantify SICI and ICF, peak-to-peak amplitudes of unconditioned and conditioned MEPs were calculated, and the conditioned MEP amplitudes were expressed as a percentage of unconditioned MEP amplitudes.

**Table 1:** Root-mean-square EMG activity (mV; mean ± SD) in the 100 ms preceding the stimulus

	Experiment 1			Experiment 2		Experiment 3			Experiment 4
	Short	Intermediate	Long	MEP	LEP	MEP	SICI	ICF	*H*-reflex
SOL									
STAT	0.0105 ± 0.0003	0.0105 ± 0.0004	0.0105 ± 0.0002	0.0107 ± 0.0002	0.0109 ± 0.0006	0.0106 ± 0.0001	0.0106 ± 0.0002	0.0106 ± 0.0001	0.0105 ± 0.0001
SHO	0.0107 ± 0.0002	0.0107 ± 0.0002	0.0108 ± 0.0002	0.0108 ± 0.0003	0.0108 ± 0.0002	0.0107 ± 0.0003	0.0107 ± 0.0001	0.0106 ± 0.0001	0.0105 ± 0.0001
LEN	0.0107 ± 0.002	0.0107 ± 0.0002	0.0107 ± 0.0002	0.0106 ± 0.0001	0.0107 ± 0.0002	0.0106 ± 0.0001	0.0106 ± 0.0002	0.0106 ± 0.0001	0.0105 ± 0.0001
TA									
STAT	0.0042 ± 0.0005	0.0042 ± 0.0004	0.0041 ± 0.0003	0.0044 ± 0.0003	0.0046 ± 0.0004	0.0042 ± 0.0002	0.0042 ± 0.0002	0.0042 ± 0.0002	0.0043 ± 0.0001
SHO	0.0044 ± 0.0006	0.0044 ± 0.0007	0.0042 ± 0.0003	0.0046 ± 0.0003	0.0046 ± 0.0002	0.0045 ± 0.0005	0.0043 ± 0.0002	0.0043 ± 0.0003	0.0043 ± 0.0001
LEN	0.0042 ± 0.0003	0.0043 ± 0.0004	0.0044 ± 0.0006	0.0046 ± 0.0002	0.0047 ± 0.0003	0.0043 ± 0.0002	0.0043 ± 0.0003	0.0043 ± 0.0002	0.0044 ± 0.0001

*SOL* soleus, *TA* tibialis anterior, *STAT* static position, *SHO* passive shortening, *LEN* passive lengthening, *MEP* motor-evoked potential, *LEP* lumbar-evoked potential, *SICI* intracortical inhibition, *ICF* intracortical facilitation

### Statistical analyses

All data are presented as mean ± SD. Normality of data was assessed using Shapiro–Wilk test. If the data were not normally distributed, transformations were performed using common logarithm. A paired-sample *T* test was used to assess differences in stimulus intensity at rMT (% of stimulator output; SO) between SOL and TA. Sphericity was assessed using Mauchly’s test of sphericity. In the case of violation, a Greenhouse–Geisser correction was employed. A repeated-measures ANOVA was used to assess differences in normalised evoked responses between resting position and passive shortening and lengthening (within-factor—a change in muscle length). Additional factor was added to ANOVA to assess differences between stimulations performed at different lengths (within-factor—muscle length at the point of stimulation). A two-way ANOVA was used to assess differences in fascicle length with passive ankle movement (2 × direction—shortening and lengthening; 5 × joint angle). If significant *F* values were found, analyses were continued using pairwise comparison with Bonferroni correction. In addition, Pearson’s class correlation and a linear regression were performed to assess the association of intracortical facilitation or inhibition to a change in MEP/*M*_max_ with a change in shortening or lengthening. Significance was set at an alpha level of 0.05. All analyses were performed using SPSS (v20, SPSS Inc., Chicago, IL, USA).

## Results

### Fascicle length changes during passive ankle movement

**Fig. 3:**
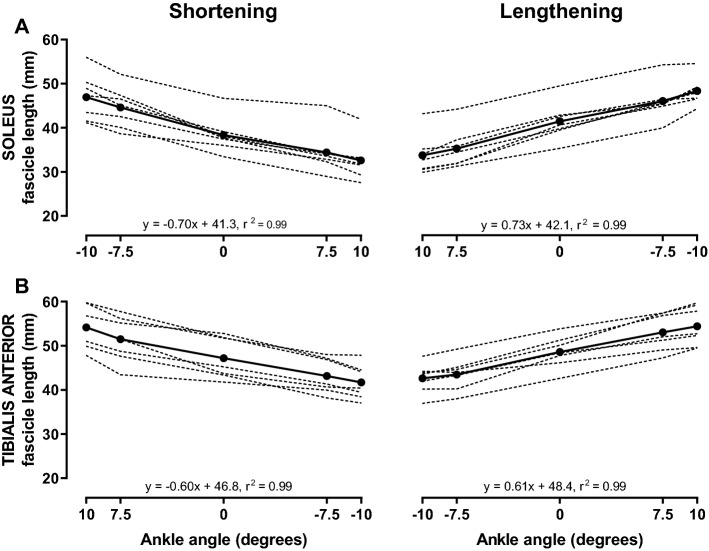
Change in fascicle length in soleus and tibialis anterior with passive movement of the ankle. Fascicle length (mm) with passive changes in the ankle joint angle during passive shortening (left panel) and lengthening (right panel) of soleus (**a**) and tibialis anterior (**b**). Fascicle length was assessed at joint angles where stimuli were delivered in subsequent experiments and are displayed on the *x*-axes relative to anatomical zero (ankle at 90°). Fascicles changed linearly with changes in joint angle as noted on plots. Full lines represent the sample mean, whilst dashed lines denote individual responses (*n* = 7)

Fascicle length was modulated during passive ankle movement both in SOL (*F*_4,24_ = 109.9, *p* < 0.001; Fig. 3a) and TA (*F*_4,24_ = 239.9, *p* < 0.001; Fig. 3b), such that fascicle length changed linearly (Fig. 3) with changes in joint angle throughout the 20° of range of motion (*p* ≤ 0.003 and *p* ≤ 0.002 for SOL and TA, respectively). Based on total change in fascicle length throughout the range of motion, the fascicles exhibited a similar mean change of 0.7 mm/° and 0.6 mm/° in SOL and TA, respectively (*p* = 0.388). In TA, fascicles were on average longer during passive lengthening (40.9 ± 4.1 mm) compared to passive shortening (39.4 ± 4.5 mm; *F*_1,6_ = 10.3, *p* = 0.018). However, no direction × angle interaction was found for both SOL (*F*_4,24_ = 1.5, *p* = 0.240) and TA (*F*_4,24_ = 1.2, *p* = 0.357).

### Experiment 1: corticospinal responses at different muscle lengths during passive ankle movement

**Fig. 4:**
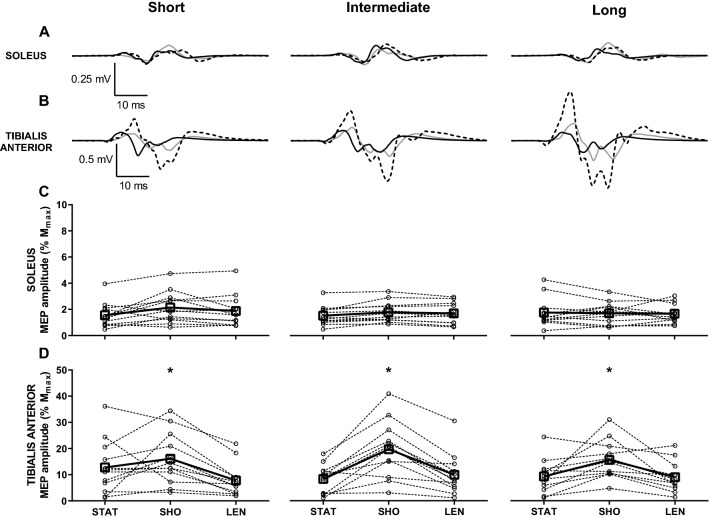
Motor-evoked potentials during static position, passive shortening and lengthening in soleus, and tibialis anterior with stimuli delivered at different muscle length. **a**, **b** Averaged representative traces in response to single-pulse transcranial magnetic stimulation delivered at short, intermediate, and long muscle length during resting position (black line), passive shortening (grey line), and lengthening (dashed line) in soleus (**a**) and tibialis anterior (**b**). Each representative trace is an average of 15 waveforms. **c**, **d** Amplitude of motor-evoked potential expressed as a percentage of the amplitude of maximal compound action potential (MEP/*M*_max_) during static position (STAT), passive shortening (SHO), and passive lengthening (LEN) in soleus (**c**) and tibialis anterior (**d**) at short (left panel), intermediate (centre panel), and long (right panel) muscle length. Open squares and full lines represent the sample mean, whilst open circles and dashed lines denote individual responses (*n* = 12). *p = 0.023 compared to static position, and *p* < 0.001 compared to passive lengthening

The stimulus intensity at rMT was higher in SOL (54 ± 8% SO) compared to TA (48 ± 7% SO; *t*_11_ = 3.0, *p* = 0.012). Examples of averaged EMG recordings in SOL (A) and TA (B) in response to single-pulse TMS are presented in Fig. 4. MEP/*M*_max_ amplitude of SOL did not differ between the static position and during passive ankle movement (2 ± 1 vs. 2 ± 1 vs. 2 ± 1% *M*_max_; *F*_2,22_ = 2.3, *p* = 0.121), irrespective of the joint angle at the point of stimulation (2 ± 1 vs. 2 ± 1 vs. 2 ± 1% *M*_max_ at short, intermediate and long muscle length, respectively; *F*_2,22_ = 0.2, *p* = 0.787; Fig. 4c). Conversely, a change in muscle length modulated MEP/*M*_max_ amplitude in TA (*F*_1.3,14.6_ = 11.3, *p* = 0.003) insofar as MEP/*M*_max_ amplitude was greater during passive shortening (17 ± 9% *M*_max_) compared to passive lengthening (9 ± 7% *M*_max_; *p* < 0.001) and static position (10 ± 8% *M*_max_; *p* = 0.023; Fig. 4d), with no difference between passive lengthening and static position (*p* = 0.99). In addition, MEP/*M*_max_ amplitude in TA was not affected by muscle length at the point of stimulation (12 ± 9 vs. 13 ± 10 vs. 11 ± 7% *M*_max_ at short, intermediate and long muscle length, respectively; *F*_2,22_ = 1.0, *p* = 0.922; Fig. 4d).

### Experiment 2: corticospinal and spinal motoneuronal responses during passive ankle movement

**Fig. 5:**
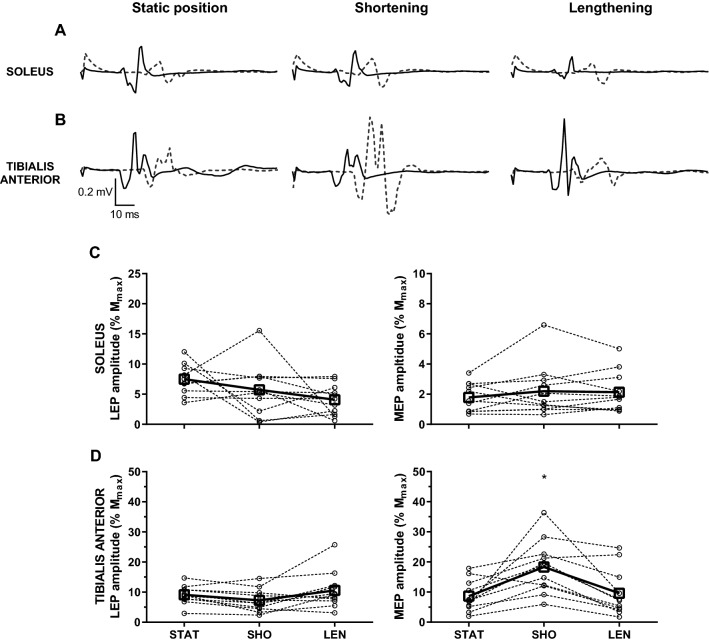
Motor-evoked and lumbar-evoked potentials during static position, passive shortening, and lengthening in soleus and tibialis anterior. **a**, **b** Averaged representative traces in response to electrical stimulation of the lumbar spinous processes (black line) and single-pulse transcranial magnetic stimulation (dashed grey line) during static position (left panel), passive shortening (centre panel) and passive lengthening (right panel) in soleus (**a**) and tibialis anterior (**b**). Each representative trace is an average of ten waveforms. **c**, **d** Amplitude of lumbar-evoked potential (left panel) and motor-evoked potential (right panel) expressed as a percentage of the amplitude of maximal compound action potential (LEP/*M*_max_ and MEP/*M*_max_, respectively) during static position (STAT), passive shortening (SHO), and passive lengthening (LEN) in soleus (**c**) and tibialis anterior (**d**). Open squares and full lines represent the sample mean, whilst open circles and dashed lines denote individual responses (*n* = 11). *p < 0.005 compared to static position and passive lengthening

The stimulus intensity at rMT was again higher in SOL (49 ± 9% SO) compared to TA (46 ± 10% SO; *t*_10_ = 3.0, *p* = 0.014). Figure 5 shows the examples of averaged EMG recordings in SOL (A) and TA (B) in response to single-pulse TMS and electrical stimulation of descending axons at the lumbar spinal segments. These responses display similarities of MEPs in SOL across conditions (Fig. 5a). Both MEP/*M*_max_ (2 ± 1 vs. 2 ± 2 vs. 2 ± 1% *M*_max_; *F*_2,20_ = 0.72, *p* = 0.497) and LEP/*M*_max_ (7 ± 2 vs. 6 ± 4 vs. 4 ± 3% *M*_max_; *F*_2,20_ = 2.95, *p* = 0.075) were not modulated in SOL during passive ankle movement (Fig. 5c). Similarly, LEP/*M*_max_ did not change in TA with passive shortening and lengthening (9 ± 3 vs. 7 ± 4 vs. 11 ± 6% *M*_max_; *F*_2,20_ = 3.63, *p* = 0.071). However, MEP/*M*_max_ was modulated by a change in muscle length in TA (*F*_2,20_ = 14.67, *p* < 0.001), being greater during passive shortening (18 ± 9% *M*_max_) compared to passive lengthening (10 ± 8% *M*_max_; *p* = 0.001) and static position (9 ± 5% *M*_max_; *p* = 0.003; Fig. 5d).

### Experiment 3: intracortical inhibition and facilitation during passive ankle movement

**Fig. 6:**
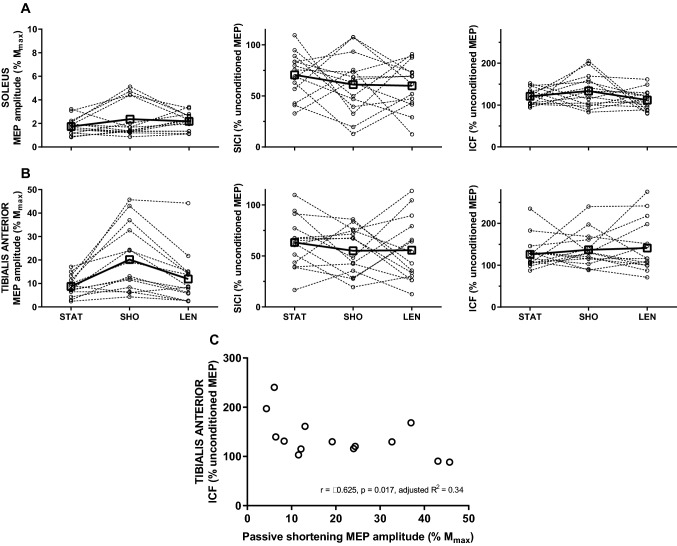
Motor-evoked potentials evoked with single- and paired-pulse transcranial magnetic stimulation during static position, passive shortening, and lengthening in soleus and tibialis anterior. **a**, **b** Amplitude of motor-evoked potential expressed as a percentage of the amplitude of maximal compound action potential (MEP/*M*_max_; left panel), short-interval intracortical inhibition (SICI; centre panel), and intracortical facilitation (ICF; right panel) expressed as a percentage of the unconditioned MEP amplitude during static position (STAT), passive shortening (SHO), and passive lengthening (LEN) in soleus (**a**) and tibialis anterior (**b**). Open squares and full lines represent the sample mean, whilst open circles and dashed lines denote individual responses (*n* = 14). *p < 0.005 compared to resting position and passive lengthening. **c** The amplitude of motor-evoked potential expressed as a percentage of the amplitude of maximal compound action potential (MEP/*M*_max_) plotted against ratio of conditioned and unconditioned motor-evoked potential amplitude (ICF) in response to paired-pulse transcranial magnetic stimulation with an inter-stimulus interval of 10 ms during passive shortening in TA (*n* = 14)

The stimulus intensity rMT was higher in SOL (51 ± 12% SO) compared to TA (48 ± 10% SO; *t*_13_ = 4.5, *p* = 0.001). MEP/*M*_max_ in SOL was not modulated with a change in muscle length (2 ± 1 vs. 2 ± 2 vs. 2 ± 1% *M*_max_; *F*_2,26_ = 1.65, *p* = 0.211; Fig. 6a), but was in the TA (*F*_2,26_ = 15.96, *p* < 0.001; Fig. 6b), such that it was greater during passive shortening (21 ± 14% *M*_max_) compared to passive lengthening (12 ± 11% *M*_max_; *p* < 0.001) and static position (9 ± 4% *M*_max_; *p* = 0.001). No modulation in SICI was observed in SOL (71 ± 22 vs. 61 ± 30 vs. 60 ± 23% unconditioned MEP; *F*_2,26_ = 0.88, *p* = 0.427) or in TA (63 ± 25 vs. 55 ± 23 vs. 56 ± 32% unconditioned MEP; *F*_2,26_ = 0.63, *p* = 0.540) during passive ankle movement, nor was ICF (SOL: 121 ± 19 vs. 134 ± 38 vs. 112 ± 24% unconditioned MEP; *F*_2,26_ = 1.85, *p* = 0.177; TA: 129 ± 37 vs. 138 ± 40 vs. 145 ± 61% unconditioned MEP; *F*_2,26_ = 0.26, *p* = 0.777). There was an inverse relationship between MEP/*M*_max_ and ICF during passive shortening of TA (*r* = − 0.625, *p* = 0.017, adjusted *r*^2^ = 0.34), suggesting that greater corticospinal excitability observed during passive shortening was associated with a smaller degree of intracortical facilitation (Fig. 6c). No other associations were found between MEP/*M*_max_ and SICI or ICF either in SOL or TA (Table 2).

**Table 2:** Associations between responses to single- and paired-pulse transcranial magnetic stimulation

	SICI (/unconditioned MEP)						ICF (/unconditioned MEP)					
	STAT		SHO		LEN		STAT		SHO		LEN	
	*r*	*p*	*R*	*p*	*r*	*p*	*r*	*p*	*r*	*p*	*r*	*p*
MEP/*M*_max_												
SOL												
STAT	− 0.160	0.584	_–_	_–_	_–_	_–_	0.138	0.637	_–_	_–_	_–_	_–_
SHO	_–_	_–_	− 0.042	0.887	_–_	_–_	_–_	_–_	− 0.143	0.626	_–_	_–_
LEN	_–_	_–_	_–_	_–_	− 0.095	0.748	_–_	_–_	_–_	_–_	− 0.301	0.296
TA												
STAT	− 0.002	0.994	_–_	_–_	_–_	_–_	− 0.284	0.326	_–_	_–_	_–_	_–_
SHO	_–_	_–_	0.077	0.794	_–_	_–_	_–_	_–_	− 0.625	0.017	_–_	_–_
LEN	_–_	_–_	_–_	_–_	− 0.319	0.267	_–_	_–_	_–_	_–_	− 0.433	0.122

*SOL* soleus, *TA* tibialis anterior, *STAT* static position, *SHO* passive shortening, *LEN* passive lengthening, *MEP/M*_*max*_ motor-evoked potential normalised to maximal compound action potential, *SICI* intracortical inhibition, *ICF* intracortical facilitation, *r* correlation coefficient, *p* significance at alpha level 0.05

### Experiment 4: *H*-reflex during passive ankle movement

**Fig. 7:**
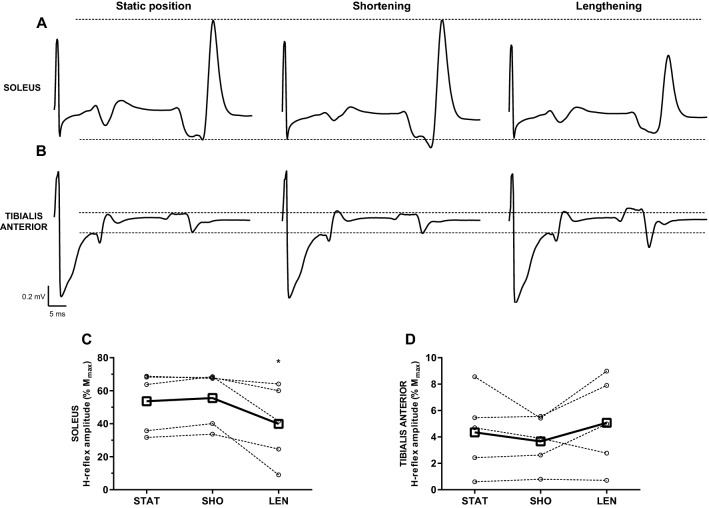
*H*-reflexes during static position, passive shortening, and lengthening in soleus and tibialis anterior. **a**, **b** Averaged representative traces in response to submaximal percutaneous nerve stimulation during static position (left panel), passive shortening (centre panel), and passive lengthening (right panel) in soleus (**a**) and tibialis anterior (**b**). Traces are shown from the point of stimulus and each representative trace is an average of ten responses. Dashed lines represent the amplitude of *H*-reflex during static position. **c**, **d** Amplitude of *H*-reflex expressed as a percentage of the amplitude of maximal compound action potential during static position (STAT), passive shortening (SHO), and passive lengthening (LEN) in soleus (**c**) and tibialis anterior (**d**). Open squares and full lines represent the sample mean, whilst open circles and dashed lines denote individual responses (*n* = 5). *p < 0.05 compared to passive shortening

Representative averaged traces of the *H*-reflex response from one individual are presented in Fig. 7a, b for SOL and TA, respectively. As clearly seen from these examples, the *H*-reflex responses were modulated during passive ankle movement in SOL (*F*_1.0,4.1_ = 8.4, *p* = 0.043), being smaller during passive lengthening (40 ± 23% *M*_max_) compared to passive shortening (56 ± 17% *M*_max_; *p* = 0.048; Fig. 7c). Conversely, *H*/*M*_max_ was not modulated during passive ankle movement in TA (4 ± 3 vs. 4 ± 2 vs. 5 ± 3% *M*_max_; *F*_2,8_ = 1.6, *p* = 0.258; Fig. 7d).

## Discussion

The main finding of this study was that corticospinal excitability is modulated differently between antagonist muscles during passive ankle movement. During passive movement, cortical excitability in TA was facilitated, but remained unchanged in SOL. Subcortical excitability at the lumbar spinal segmental level was not modulated in TA, suggesting a cortical and/or propriospinal contribution to the observed facilitation. These findings suggest a different intrinsic modulation of antagonist ankle muscles during passive movement.

### Modulation of corticospinal excitability during passive movement is not dependent on the muscle length at the point of stimulation

The differing corticospinal response to TMS between the muscles cannot be attributed to muscle length change differences, as both muscles exhibited a similar range of fascicle shortening and lengthening during passive movement (0.7 and 0.6 mm/° for SOL and TA, respectively). Contrary to our hypothesis, the responses were similar regardless of the muscle length at the point of stimulation. This contrasts also to the previous experiments of passive wrist movement (Lewis et al. 2001; Lewis and Byblow 2002) where corticospinal excitability was dependent on joint angle. However, direct comparison with the previous experiments is difficult, due to differences in muscles tested (upper limb vs. lower limb), ranges of motion, and different methodologies with regard to MEP amplitude normalisation. The latter might play a role in interpreting changes in response amplitude, since electrode position variations might lead to differences in the spatial relationship between the electrode and the motor units recorded (Farina et al. 2014), which is typically reflected in *M*_max_ amplitude (Gerilovsky et al. 1989). The range of motion, and the resultant muscle length changes, could be equally important in interpreting response amplitude. Indeed, a recent study in active knee extensors showed muscle length-dependent modulation of corticospinal excitability during lengthening contractions (Doguet et al. 2017). However, there was ~ 11 mm fascicle length change when moving from an intermediate to long position (Doguet et al. 2018), compared to ~ 5 mm seen in the present study. Thus, it seems plausible that there is a threshold of muscle length change after which increased afferent feedback is sufficient for detecting differences in corticospinal excitability.

### The responses to passive ankle movement are muscle specific

The facilitation in corticospinal response to TMS observed in TA during shortening is in agreement with studies employing passive movement in the upper limb muscles (Lewis et al. 2001; Lewis and Byblow 2002; Coxon et al. 2005; Chye et al. 2010). As LEPs and *H*-reflexes remained unchanged in TA, this would suggest a cortical and/or propriospinal origin of facilitation. Conversely, corticospinal excitability in SOL remained unchanged with passive movement. Due to lack of published data on corticospinal excitability during passive movement in SOL, no comparison can be made with the other studies. However, similar results have been obtained during active movement of SOL with comparable stimulus intensities (Duclay et al. 2011; Hahn et al. 2012; Valadão et al. 2018). Whilst LEPs remained unchanged in SOL, *H*-reflexes were reduced during passive lengthening. This latter finding corroborates the previous studies (Pinniger et al. 2001; Duclay et al. 2011), and has been attributed to presynaptic inhibition and post-activation depression of Ia afferents (Hultborn et al. 1996). Given that LEPs are likely devoid of presynaptic influence (Nielsen and Petersen 1994), the lack of LEP modulation in SOL during passive movement further corroborates the notion that presynaptic inhibition mediates the reduction in *H*-reflexes during passive lengthening.

### The activity of intracortical neurons during passive ankle movement

The MEP/*M*_max_ facilitation observed in TA during passive shortening was not accompanied by changes in responses to paired-pulse TMS, and could be explained by greater response variability (see Fig. 6). This is a common occurrence and might be due to different electrophysiological properties of neuronal populations subserving the responses to SICI and ICF and inter-individual differences in synaptic efficacy of inhibitory or excitatory interneurons (Orth et al. 2003). It was also shown that the size of the MEP/*M*_max_ during passive dorsiflexion negatively correlated with the ICF ratio, possibly due to the ‘busy line’ phenomenon, whereby glutamatergic circuitry activity is too high for conditioned MEPs to be facilitated (Ortu et al. 2008). Previous work has shown that SICI is modulated during passive wrist movements (Lewis et al. 2001), but is only evident at the transition from extension to flexion, and might be related to a sudden muscle length change and the corresponding initial burst in muscle spindle firing (Matthews 2011). When comparing responses elicited at similar joint angles, the lack of change in SICI corroborates the finding of the previous work (Lewis et al. 2001). Thus, the present data suggest that passive muscle length changes do not modulate cortical interneuronal activity.

### Cortical and propriospinal contribution to the observed corticospinal response

Increased corticospinal excitability during passive shortening in TA in the absence of LEP modulation suggests a cortical origin, associated with sensory feedback influencing the excitability of descending tracts (Meinck and Piesiur-Strehlow 1981; Roy and Gorassini 2008), or mediation via propriospinal inputs (Meinck and Piesiur-Strehlow 1981; Bestmann and Krakauer 2015).

In both primates (Hore et al. 1976; Herter et al. 2015) and humans (Goldring and Ratcheson 1972; Shaikhouni et al. 2013), cortical neurons have been shown to be facilitated during passive shortening, whilst inhibited during passive lengthening, which agrees with our findings. Cutaneous and joint receptors are unlikely mediators of this behaviour due to their activation being largely restricted to the limits of movement (Burke et al. 1988), rather than throughout the movement. Thus, the primary candidates for the sensory mediated change in cortical neuronal activity are muscle spindle afferents. This mediation might involve inhibitory inputs, either directly to motor cortical areas or through the somatosensory cortex. Indeed, in primates, hindlimb muscle stretch has been shown to result in inhibition of area 4 cortical neurons due to direct input from group II afferents (Hore et al. 1976). Furthermore, changes in TA muscle fascicle length have been shown to be tightly linked to Ia afferent sensitivity in humans (Day et al. 2017). Thus, increased corticospinal responses during passive shortening of TA might stem from decreased Ia afferent input via area 3a of the cerebral cortex (Hore et al. 1976), resulting in disinhibition of corticospinal neurons, and, thus, increasing corticospinal excitability (Brasil-Neto et al. 1992; Ziemann et al. 1998).

It is unclear why the augmented corticospinal response to TMS during passive shortening is specific to TA. It might stem from divergent, non-uniform distribution of direct corticomotoneuronal projections, as evidenced by short latency facilitation of firing probability of TA motor units in response to TMS, and the absence of this behaviour in SOL (Brouwer and Ashby 1992; Brouwer and Qiao 1995). This could have contributed to the facilitation of TA during passive shortening when corticospinal neurons may be disinhibited relative to passive lengthening (Brasil-Neto et al. 1992; Ziemann et al. 1998). There is some basis for this notion as greater facilitation during passive shortening has been observed in the wrist muscles with greater strength of corticomotoneuronal projections (Chye et al. 2010). The pyramidal tract also has a preferential input into the spinal network controlling ankle flexors, such as TA (Brooks and Stoney 1971), which could explain the lower stimulus intensity at rMT in the present study whilst also supporting the previous work (Lauber et al. 2018). In addition, the responses in TA could be related to differing reciprocal inhibition compared to SOL (Yavuz et al. 2018). Less reciprocal inhibition as SOL lengthens would suppress the excitatory postsynaptic potential stemming from the antagonist, allowing for reduced inhibition in corticospinal neurons in TA. Furthermore, as per *H*-reflex behaviour in the present study, TA appears to be influenced by presynaptic inhibitory mechanisms to a lesser extent than SOL. Thus, the facilitation observed during passive shortening of TA could be due to coupling of the lack of presynaptic influences and sensory-related facilitation of corticospinal excitability in response to movement (Schubert et al. 1997). Nonetheless, it should be noted that despite the plausibility of the above-mentioned notions, this study cannot directly ascertain the mechanism of the observed behaviour.

An increase in presynaptic inhibitory input to alpha motoneurons was observed during passive lengthening of SOL, with no accompanying change in MEPs and LEPs. This suggests a form of compensatory action of descending pathways during passive SOL lengthening to accommodate for reduced motoneuronal excitability. Given a lack of change in ICF and SICI, this compensation is unlikely to be intracortical in origin, pointing to the possibility of propriospinal mediation. This could occur through facilitation of excitatory premotoneurons activated by group II afferents (Marque et al. 2005), which are likely to exhibit increased firing rate during muscle lengthening (Matthews 2011). The specificity of this compensation to SOL is less clear, but it might again be related to asymmetrical distribution of reciprocal inhibitory input between TA and SOL (Yavuz et al. 2018).

### Potential functional applications of the observed behaviour

The specificity of augmented corticospinal response in TA relative to SOL during passive shortening could reflect functional differences between these muscles. For example, during quiet standing, TA has been shown to exhibit passive fascicle length changes proportional to the sway-related changes in the ankle joint (Di Giulio et al. 2009; Day et al. 2013). The present data might, thus, suggest an important role of increasing corticospinal drive in this muscle during passive shortening when proprioceptive feedback originating from muscle spindles is reduced, to modulate the control signals of the antagonist via reciprocal inhibition (Di Giulio et al. 2009; Honeycutt et al. 2012).

### Methodological considerations

The lack of modulation of corticospinal excitability during passive movement of the ankle in SOL could be due to the slow movement velocity used in the present study. Indeed, the previous work using higher movement velocities has shown greater modulation in response size (Lewis et al. 2001; Lewis and Byblow 2002), likely due to higher afferent feedback. The slower velocity was employed to ensure greater ability of relaxation and to avoid reflexive muscle activity related to passive movement, which could have confounded results (Pinniger et al. 2001). Furthermore, the relatively smaller ankle range of motion in the present experiment reflects the restriction and variability in joint mobility, particularly at dorsiflexion. In the upper limb, the previous research has shown potentiated effects on corticospinal excitability during passive movement with greater ranges of motion (Coxon et al. 2005). Thus, future studies should explore the velocity- and muscle-length dependence of the responses.

Other limitations of the present study are the lack of repeated-measures design and a small sample size in Experiment 4. With regard to the former, the significant facilitation of the response to TMS during passive shortening of TA was replicated across three experiments (Experiment 1–3), suggesting a universal behaviour across different sample populations. As already noted, there was difficulty in obtaining *H*-reflexes in resting TA, corroborating the previous reports (Roy and Gorassini 2008; Burke 2016). Despite screening 24 individuals, only five participants exhibited consistent *H*-reflexes in TA to allow for comparison with SOL. This small sample size does warrant caution in interpreting the findings of Experiment 4. However, the SOL data corroborates the findings of previous work (Pinniger et al. 2001) and suggests that presynaptic inhibition during passive lengthening is greater compared to TA.

## Conclusions

As hypothesised, the segmental methodological approach revealed that changing muscle length modulates both corticospinal and spinal elements of the nervous system during passive movement, but is muscle specific. Contrary to our hypothesis, the corticospinal modulation occurred regardless of the muscle length at the point of assessment. Corticospinal excitability was facilitated in TA during passive shortening, whilst unmodulated in SOL. This suggests that neural modulation with movement should be interpreted in the context of the muscle investigated. During muscle shortening, a reduced inhibitory afferent input might explain the flexor-biased facilitation in corticospinal drive.
